# Associations between Extracellular Matrix Protein 1 Gene Polymorphism and Progression of Liver Disease

**DOI:** 10.1155/2022/9304264

**Published:** 2022-10-14

**Authors:** Xiuting He, Ting Liu, Rui Zhang, Xu Li

**Affiliations:** ^1^Departments of Geriatrics, The First Hospital of Jilin University, Changchun, Jilin, China; ^2^Departments of Comprehensive Ultrasonography, The First Hospital of Jilin University, Changchun, Jilin, China; ^3^Departments of Hepatology, The First Hospital of Jilin University, Changchun, Jilin, China

## Abstract

**Background:**

Our study aimed to investigate the relationship between extracellular matrix 1 (*ECM1)* gene polymorphism and progression of liver fibrosis in the Chinese population.

**Methods:**

A total 656 patients with hepatitis B virus (HBV) infection and 298 healthy individuals of the Chinese Han population were recruited for a retrospective case-control study. Of the disease group, 104 cases had chronic hepatitis B (CHB), 266 had LC, and 286 had hepatocellular carcinoma (HCC). Subjects were frequency-matched according to age and gender. Polymorphisms of the *ECM1* gene were examined using the MassARRAY SNP genotyping method.

**Results:**

There were no associations between genotype and allele frequencies of *ECM1* rs3737240 and rs13294 loci with the risk of CHB and CHB-related HCC. After adjustment for age, sex, smoking status, and drinking habits, the GT genotype was dramatically related to a reduced risk of chronic HBV infection in both non-HCC (OR = 0.68, 95% CI: 0.49-0.94) and total chronic HBV infection patients (OR = 0.75, 95% CI: 0.56-1.00). Haplotype analyses revealed twelve protective haplotypes against total chronic HBV infection and four against non-HCC chronic HBV infection.

**Conclusion:**

*ECM1* gene polymorphism in rs3834087 and rs3754217 loci is associated with a reduced risk of chronic HBV infection but not with liver fibrosis development and the occurrence of HCC.

## 1. Introduction

Approximately 240 million people, especially those in developing countries, are infected with hepatitis B virus (HBV), and nearly half of these have chronic liver disease [1, 2]. Liver fibrosis is a pathological consequence of chronic liver damage and extracellular matrix (ECM) protein accumulation, which may further result in liver diseases such as liver cirrhosis (LC), liver failure, and hepatocellular carcinoma (HCC) [3–5]. Liver fibrosis and its associated liver diseases have been considered the most important causes of HBV-induced death [6]. Although the molecular mechanisms of liver fibrosis have been well documented, more effective drugs for liver fibrosis need to be explored [7, 8].

Regardless of whether a toxic or metabolic pathway ensues, a hepatitis virus infection involves the induction of immune cell infiltration and hepatocyte injury, triggering the trans-differentiation of hepatic stellate cells (HSCs) into collagen-producing myofibroblasts [9, 10], and causing deposition of ECM which leads to liver tissue damage [11, 12]. Human *ECM1* gene, located on chromosome 1q21 [13, 14], encodes for a glycoprotein which promotes ECM protein binding and regulates the integrity and barrier function of epidermal ECM in a variety of tissues [13, 15, 16]. A recent study on patients and mouse models has shown an inverse correlation between the *ECM1* production in chronic hepatitis liver tissues with transforming growth factor-*β* activation which is responsible for promoting fibrosis and inducing the activation of static HSCs [17].

Several studies identified genetic variants that are associated with inflammatory bowel disease, such as ulcerative colitis [18, 19] and Crohn's disease [20]. However, the correlation between *ECM1* gene polymorphisms and fibrogenic disease formation has not been reported. In this study, we screened the *ECM1* gene polymorphisms of both regulatory and coding regions that could modify *ECM1* transcription or amino acid sequence and analyzed their association with LC progression in Chinese Han population. Our study might aid in understanding the effects of *ECM1* gene polymorphism on the initiation and progression of liver fibrosis and on the occurrence of HCC.

## 2. Methods

### 2.1. Subjects

A total of 656 patients with chronic HBV infection, diagnosed as chronic hepatitis B (CHB) (*n* = 104), LC (*n* = 266), and HCC (*n* = 286) based on the Guideline of Prevention and Treatment for CHB [21], were enrolled from the First Hospital of Jilin University [22]. Patients within each group were frequency-matched based on age and sex. CHB was defined as a persistent or intermittent elevation of serum alanine aminotransferase (ALT) (≥2xUTL) level, with increased HBV-DNA level (≥500 IU/ML) for at least 6 months, and the absence of hepatitis C virus (HCV) and human immunodeficiency virus (HIV) infections. CHB/LC was diagnosed as active necroinflammatory liver disease with/without fibrosis by imageological inspection. The diagnosis of HCC was confirmed with histopathology. The exclusion criteria involved the presence of HAV/HEV infection, autoimmune diseases, alcoholic liver disease, silt hemorrhagic liver disease, autoimmune liver disease, and intra- and extrahepatic bile duct stones. Another 298 liver disease-free individuals were selected as healthy controls, and they were negative for HBeAg, HBsAg, anti-HBc, anti-HBe, and anti-HCV. Demographic information, including drinking habits and smoking status, was collected for analysis. Drinkers were classified as those who consumed alcohol more than once per week for more than six months, while smokers were those who smoke daily for over a year. All subjects provided informed consent, and the research was approved by the ethical committee of The First Hospital of Jilin University (Approval number: 2020-Linshen No. 2020-355). All methods were carried out in accordance with relevant guidelines and regulations.

### 2.2. SNP Selection

**Figure 1:**
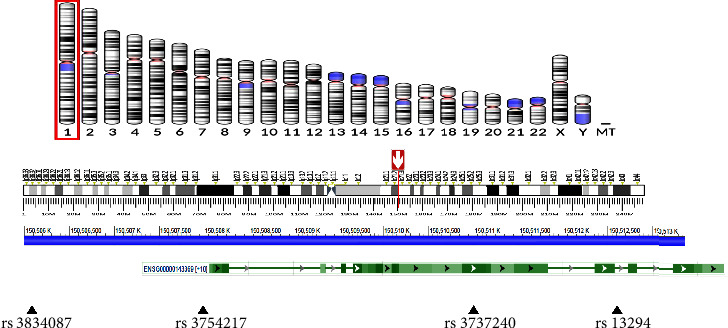
Location of the extracellular matrix 1 gene and single nucleotide polymorphisms.

SNPs from the promoter, 5′ untranslated regions (UTRs), exons, and 3′ UTR of the *ECM1* gene (minor allele frequency [MAF] >5% in the Northern Chinese Han population database) were screened using GeneView of NCBI. SNP function prediction was carried out using the online tool (https://snpinfo.niehs.nih.gov/). Linkage disequilibrium (LD) analysis was performed for the selected SNPs using the “Linkage Disequilibrium Calculator” (https://asia.ensembl.org/Homo_sapiens/Tools/LD?db=core). One of the complete linkage sites were chosen if *R*2 = 1. Validated and hot SNPs with reported relationship with the disease on Google Scholar were also selected and verified for MAF >5% in Beijing Chinese Han population. Of the SNPs found in the promoter region (rs3834087 and rs3754217), the latter has been predicted to be located in the transcription factor binding site (TFBS) and may have effects on genetic transcription. SNPs found in the exon-missense region (rs3737240 and rs13294) may affect the expression and function of the gene. The *ECM1* gene location and the selected SNPs are exhibited in Figure 1.

### 2.3. Genotype Analysis

Genomic DNA was isolated from whole blood of subjects. SNP genotypes of rs3834087, rs3754217, rs3737240, and rs13294 were analyzed by using the MassARRAY SNP genotyping method (BioMiao Biological Technology, Beijing, CHN). Polymerase chain reaction (PCR) primers and the amplification program are shown in Table 1.

### 2.4. Statistical Methods

All data were analyzed using the SPSS 18.0 software (SPSS, Chicago, USA). The differences in clinical data and demographic information among groups were calculated using *χ*^2^ test or Wilcoxon rank-sum test. Independent segregation of alleles was tested by the Hardy–Weinberg equilibrium (H-WE) analysis. Haplotype analysis of polymorphism was performed by using the UNPHASED 3.1.4 software. *P*-value, odds ratios (ORs), and 95% confidence intervals (CIs) after adjusting for age, gender, and environmental factors were calculated by the bivariate logistic regression analysis. Genotype distributions and allele frequencies were analyzed by the *χ*2 test or Fisher's exact test. *P*-value < 0.05 indicated statistical significance.

## 3. Results

Demographic information of patients including gender, smoking status, age, and alcohol intake is summarized in Tables 2 and 3. No statistical differences were found in gender (*χ*^2^ test) and age (Wilcoxon rank-sum test) among the groups (*P* > 0.05) except between non-HCC and HCC groups (*P*=0.0482 and *P*=0.0220, respectively). However, significant differences were observed in smoking status (*χ*2 test) between HCC and healthy controls (*P*=0.0023), HCC and LC (*P*=0.0001), as well as HCC and non-HCC (*P* < 0.0001). The same was observed in drinking habits (*χ*2 test) between non-HCC and healthy controls (*P*=0.0416) and non-HCC and HCC (*P*=0.0229). Furthermore, the H-WE test determined that all four SNPs (rs3834087, rs3754217, rs3737240, and rs13294) of the controls were in equilibrium (*P*=0.0916, *P*=0.0963, *P*=0.5324, and *P*=0.7512, respectively).

### 3.1. Analysis in Chronic HBV Infection Patients and Healthy Controls

The allele and genotype frequencies of the *ECM1* SNPs in HBV infected-patients and the control group are displayed in Table 3. There were no correlations between the allele and genotype frequencies of the *ECM1* gene polymorphisms at rs3737240 and rs13294 loci of patients with chronic HBV infection. There was an obvious association between the GAG/- genotype of rs3834087 with reduced risk of chronic HBV infection (OR = 0.65, 95% CI: 0.45-0.95). After adjustment for age, smoking status, sex, and drinking habits, bivariate logistic regression analyses showed that the GAG/- and -/- combined genotypes were markedly correlated with a declined risk of chronic HBV infection (OR = 0.66, 95% CI: 0.45-0.96). The GT genotype of rs3754217, as compared to the wild GG genotype, was closely related to a reduced risk of chronic HBV infection (adjusted OR = 0.75, 95% CI: 0.56-1.00).

### 3.2. Analysis in HCC Patients and Healthy Controls

The genotype and allele frequencies of the *ECM1* SNPs in HCC patients and the control group are presented in Table 4. There were no differences in the frequencies of all alleles and genotypes (rs3834087, rs3754217, rs3737240, and rs13294).

### 3.3. The Genotype and Allele Frequencies of *ECM1* SNPs in Non-HCC Patients

The genotype and allele frequencies of *ECM1* SNPs in non-HCC patients and the control group are presented in Table 4. No obvious effects were identified between the genotype and allele frequencies of *ECM1* SNPs at rs3737240 and rs13294 loci and in the risk of non-HCC after adjusting for smoking status, drinking habits, sex, and age.

However, the DEL allele of rs3834087 was markedly correlated with a decreased risk of CHB (OR = 0.60, 95% CI: 0.39-0.92). Compared to GAG/GAG, the GAG/- and -/- genotypes of rs3834087 were greatly associated with a decreased risk of CHB (OR = 0.54, 95% CI: 0.35-0.85; and OR = 0.55, 95% CI: 0.35-0.86, respectively). Additionally, with adjustment for age, sex, smoking status, and drinking habits, the GT and GT plus TT genotypes of rs3754217, when compared to the wild GG genotype, correlated with a reduced risk of CHB (OR = 0.68, 95% CI: 0.49-0.94; OR = 0.71, 95% CI: 0.52-0.98, respectively).

### 3.4. Distribution Difference between HBV Infection Subgroups

The allele and genotype frequencies of the *ECM1* SNPs in non-HCC (including CHB and LC) and HCC patients are summarized in Table 5. There were no differences in the frequencies of all alleles and genotypes (rs3834087, rs3754217, rs3737240, and rs13294) between the LC and CHB, LC and HCC, and non-HCC and HCC patients.

### 3.5. Haplotype Analysis

The haplotype distributions of healthy individuals against those of chronic HBV infection, non-HCC, and HCC patients were analyzed, and positive results are shown in Table 6. Between chronic HBV infection patients and controls, the following haplotypes greatly correlated with a decreased risk of chronic HBV infection when compared to their wild type alleles: G-T and T-C alleles of rs3754217-rs3737240 (OR = 0.54, 95% CI: 0.31-0.96; OR = 0.64, 95% CI: 0.44-0.93, respectively) and G-G-T and Del-T-C alleles of rs3834087-rs3754217-rs3737240 (OR = 0.54, 95% CI: 0.31-0.96; OR = 0.65, 95% CI: 0.45-0.96, respectively).

Between non-HCC chronic HBV infection patients and healthy controls, the following haplotypes were related to a decreased risk of non-HCC chronic HBV infection: the T-C alleles of rs3754217-rs3737240 (OR = 0.51; 95% CI: 0.33-0.81) and Del-T alleles of rs3834087-rs3754217 (OR = 0.61; 95% CI: 0.40-0.95) (Table 7).

Liver stiffness measurement (LSM, kPa) scores collected using FibroScan on CHB patients with the different genotypes of rs3834087 and rs3754217 are shown in Supplemental Table 1. Patients with the GT and the GT plus TT genotypes demonstrated lower LSM scores as than that of wild GG genotype patients (Supplemental Table 1). No significant differences were found in the Child-Pugh scores of LC between the different genotypes (Supplemental Table 2).

## 4. Discussion

Mutation of *ECM1* may lead to various genetic diseases such as lipoid proteinosis and autosomal recessive genodermatosis [23, 24]. *ECM1* has also been identified to be involved in the differentiation and function of immune cells. Its important role in fibrosis was only recently discovered, but published results have been controversial. In a research by Fan et al. on liver fibrosis in mouse models, liver damage was shown to reduce the levels of *ECM1* production during fibrogenesis, and re-expression of *ECM1* prevented liver fibrosis progression [17]. In contrast, another study on heart fibrosis showed that *ECM1* led to cardiac fibrosis in myocardial infarction by acting as an intermediary between inflammation and fibrosis [25]. We therefore conducted this large case-control study to evaluate the effects of *ECM1* on the progression of liver fibrosis *in vivo*. To our knowledge, our study is the first to have investigated the relationships between *ECM1* SNPs and the development of chronic liver fibrosis; other articles on *ECM1* SNPs have only involved extrahepatic diseases [18, 20, 26, 27].

We found that the GAG/- and GAG/- plus -/- genotypes of rs3834087 were correlated with a reduced risk of chronic HBV infection both in non-HCC (OR = 0.54, 95% CI: 0.35-0.85; OR = 0.55, 95% CI: 0.35-0.86, respectively) and total chronic HBV infection patients (OR = 0.65, 95% CI: 0.45-0.95; OR = 0.66, 95% CI: 0.45-0.96, respectively). The Del allele of rs3834087 was also related to a lower risk of non-HCC chronic HBV infection (OR = 0.60, 95% CI: 0.39-0.92). After adjustment for age, sex, smoking status, and drinking habits, the GT genotype of rs3754217 was significantly related to a declined risk of chronic HBV infection both in non-HCC (OR = 0.68, 95% CI: 0.49-0.94) and in total chronic HBV infection patients (OR = 0.75, 95% CI: 0.56-1.00). The GT plus TT genotype of rs3754217 was also related to a decreased risk of non-HCC chronic HBV infection (OR = 0.71, 95% CI: 0.52-0.98). Haplotype analyses showed significant association of several haplotypes with a reduced risk of chronic HBV infection, including the T-C allele of rs3754217-rs3737240, DE-T-G allele of rs3834087-rs3754217-rs13294, and the T-C-G allele of rs3754217-rs3737240-rs13294. The Del allele of rs3834087 and the T allele of rs3754217 may be the influencing factors of chronic HBV infection. The mechanisms of these genotype differences on HBV susceptibility remain unclear and warrant further investigations. The rs3834087 and rs3754217 loci in the promoter region of *ECM1* and the rs3754217 locus initially predicted as the TFBS were speculated to affect the functions of other associated genes. Previous studies have found that the rs3737240 and rs13294 loci exhibited strong associations with the occurrence of ulcerative colitis [20]. However, we did not find the role of these two SNPs in chronic hepatitis B occurrence, progression of liver fibrosis, and HCC.

In the analysis of the subgroups, no effects on the progression from chronic hepatitis to cirrhosis and HCC were found in the four SNP variants (rs3834087, rs3754217, rs3737240, and rs13294). However, LSM, which has displayed excellent diagnostic accuracy in the identification of HBV-associated fibrosis and cirrhosis [6, 28], revealed significant differences between the GT, GT plus TT, and wild TT genotypes of rs3754217. Mutant genotypes showed low LSM scores, which reflected the lower degree of fibrosis. Due to the limited samples, further studies with expanded sample number are required for further confirmation.

## 5. Limitations

The primary limitation of this study is that this case-control study was a hospital-based study. Thus, selection bias may have occurred. In addition, due to technical problems in functional studies, we did not find direct evidence on whether these two polymorphisms (rs3834087 and rs3754217) located in promoter regulate or influence *ECM1* expression. Therefore, further functional studies are warranted.

## 6. Conclusion

In conclusion, our study preliminarily demonstrated that the *ECM1* locus may mediate the chronicity of HBV infections *in vivo*. The mechanisms involved in inflammation and fibrosis should be further explored. It should be noted that identification of new therapeutic targets is still required to promote the development of new antifibrotic drugs and fibrotic biomarkers to improve the management of fibrosis.

## Figures and Tables

**Table 1:** Primer sequences and reaction conditions for genotyping ECM1 polymorphisms.

SNP	Sequence of the primer	Annealing temperature (°C)
Rs3834087	F:5′-ACGTTGGATGAGACCTAGATGGAATCAGCC-3′	47.9
	R:5′-ACGTTGGATGTGAAAAAGGGAGCATGGCAG-3′	

Rs3754217	F:5′ACGTTGGATGGGGACTGATTAGAGGAGAAC-3′	55.5
	R:5′-ACGTTGGATGAACTGAGGCACAAACTAGGG-3′	

Rs3737240	F:5′-ACGTTGGATGTGTGGGTTCCTTCACATGTC-3′	50.8
	R:5′-ACGTTGGATGATGGCTCTGGTCCCCAAATG-3′	

Rs13294	F:5′-ACGTTGGATGCTCTTTGGTTTCCACAGAGG-3′	51.6
	R:5′-ACGTTGGATGTATGACCGGGACATCTTGAC-3′	

**Table 2:** Baseline characteristics of 954 study subjects.

Group	Healthy control *n* = 298	Chronic HBV infection patient				*P -value* ^*c*^
		Non-HCC		HBV-related HCC		
		*n* = 370	*P -value* ^*a*^	*n* = 286	*P -value* ^*b*^	
Male, *n* (%)	246 (82.6)	295 (79.7)	0.3734	245 (85.7)	0.3112	1.0000
Age, M (P25, P75)	50 (45,55)	49 (42,55)	0.0885	50 (46,56)	0.5214	0.4585

Smoking			0.4654		*0.0023*	0.2574
Ever, *n* (%)	112 (37.6)	128 (34.6)		144 (50.3)		
Never, *n* (%)	186 (62.4)	242 (65.4)		142 (49.7)		

Drinking			*0.0416*		0.8052	0.2542
Ever, *n* (%)	121 (40.6)	122 (33.0)		119 (41.6)		
Never, *n* (%)	177 (59.4)	248 (67.0)		167 (58.4)		

*P*-value^a,b,c^ represents the non-HCC, HCC, and chronic HBV patients compared to the healthy controls. The rank-sum test or *χ*2 test was used to evaluate continuous numeric variables and grouping variables in demographic data among the groups. HBV, hepatitis B virus; HCC, hepatocellular carcinoma.

**Table 3:** Baseline characteristic of chronic HBV infection subgroups.

	Non-HCC			HCC		
	CHB *n* = 104	*P -value* ^*a*^	LC *n* = 266	*P -value* ^*b*^	*n* = 286	*P -value* ^*c*^
Male, *n* (%)	84 (80.8)	0.7558	211 (79.3)	0.0495	245 (85.7)	*0.0482*
Age, M (P25, P75)	47 (43,53)	0.3404	49 (41,56)	0.1443	50 (46,56)	*0.0220*

Smoking		0.8039		*0.0001*		*<0.0001*
Ever, *n* (%)	37 (35.6)		91 (34.2)		144 (50.3)	
Never, *n* (%)	67 (64.4)		175 (65.8)		142 (49.7)	

Drinking		0.1217		0.1305		*0.0229*
Ever, *n* (%)	28 (26.9)		94 (35.3)		119 (41.6)	
Never, *n* (%)	76 (73.1)		172 (64.7)		167 (58.4)	

*P*-value^a,b,c^ represents the CHB *vs.* LC, LC *vs.* HCC, and non-HCC *vs.* HCC, respectively. The rank-sum test or *χ*2 text was used to evaluate continuous numeric variables and grouping variables in demographic data among the groups. CHB, chronic hepatitis B; CI, confidence interval; HBV, hepatitis B virus; HCC, hepatocellular carcinoma; LC, liver cirrhosis; OR, odds ratio.

**Table 4:** Genotype and allele frequencies of SNPs in the *ECM1* gene in each group.

SNP	Healthy control	Chronic HBV infection patient						Chronic HBV infection patient (*N* = 656) vs. healthy control (*N* = 298)	
		Non-HCC (*N* = 370)			HBV-related HCC (*N* = 286)				
	*N* (%)	*N* (%)	OR (95% CI)	*P -value* ^*a*^	*N* (%)	OR (95% CI)	*P*-value^b^	OR (95% CI)	*P*-value^c^
*Rs3834087 genotype and allele*									
Detected number	*N* = 298	*N* = 370			*N* = 286			*N* = 656 vs. *N* = 286	
GAG/GAG	245 (82.2)	330 (89.2)	1		243 (85.0)	1		1	
GAG/-	53 (17.8)	39 (10.5)	0.54 (0.35-0.85)	*0.0068*	43 (15.0)	0.79 (0.50-1.23)	0.2921	0.65 (0.45-0.95)	0.0248
-/-	0 (0.0)	1 (0.3)	—	1.0000	0 (0.0)	—	—	—	1.0000
GAG/- + -/-	53	40	0.55 (0.35-0.86)	*0.0089*	43	0.79 (0.50-1.23)	0.2923	0.66 (0.45-0.96)	0.0287
GAG allele	543 (91.1)	699 (94.5)	1		529 (92.5)	1		1	
DEL allele	53 (8.9)	41 (5.5)	0.60 (0.39-0.92)	*0.0172*	43 (7.5)	0.83 (0.55-1.27)	0.3922	0.70 (0.49-1.00)	0.0514

*Rs3754217 genotype and allele*									
Detected number	*N* = 294	*N* = 369			*N* = 284			*N* = 653 vs. *N* = 294	
GG	144 (49.0)	210 (56.9)	1		146 (51.4)	1		1	
GT	132 (44.9)	134 (36.3)	0.68 (0.49-0.94)	*0.0209*	114 (40.1)	0.83 (0.59-1.17)	0.2782	0.75 (0.56-1.00)	0.0481
TT	18 (6.1)	25 (6.8)	0.95 (0.50-1.82)	0.8850	24 (8.5)	1.31 (0.68-2.55)	0.4180	1.11 (0.63-1.98)	0.7152
GT + TT	150	43	0.71 (0.52-0.98)	*0.0351*	138	0.88 (0.63-1.23)	0.4642	0.79 (0.60-1.04)	0.0981
G allele	420 (71.4)	554 (75.1)	1		406 (71.5)	1		1	
T allele	168 (28.6)	184 (24.9)	0.83 (0.65-1.06)	0.1358	162 (28.5)	1.00 (0.77-1.29)	0.9847	0.90 (0.73-1.12)	0.3469

*Rs3737240 genotype and allele*									
Detected number	*N* = 296	*N* = 369			*N* = 286			*N* = 655 vs. *N* = 296	
CC	176 (59.5)	224 (60.7)	1		169 (59.3)	1		1	
CT	102 (34.5)	127 (34.4)	0.96 (0.69-1.34)	0.8270	100 (35.1)	1.02 (0.71-1.44)	0.9335	0.99 (0.74-1.33)	0.9441
TT	18 (6.1)	18 (4.9)	0.79 (0.40-1.57)	0.4982	16 (5.6)	0.91 (0.47-1.86)	0.7979	0.85 (0.47-1.56)	0.6069
CT + TT	120	145	0.94 (0.68-1.29)	0.6870	116	1.00 (0.71-1.40)	0.8231	0.97 (0.73-1.28)	0.8282
C allele	454 (76.7)	575 (77.9)	1		438 (76.8)	1		1	
T allele	138 (23.3)	163 (22.1)	0.94 (0.64-1.37)	0.7415	132 (23.2)	1.00 (0.76-1.31)	0.9783	0.98 (0.76-1.26)	0.8509

*Rs13294 genotype and allele*									
Detected number	*N* = 297	*N* = 369			*N* = 285			*N* = 654 vs. *N* = 297	
GG	176 (59.3)	224 (60.7)	1		169 (59.3)	1		1	
GA	104 (35.0)	127 (34.4)	0.94 (0.68-1.31)	0.7223	100 (35.1)	1.00 (0.70-1.24)	0.9969	0.97 (0.72-1.30)	0.8412
AA	17 (5.7)	18 (4.9)	0.84 (0.42-1.68)	0.6120	16 (5.6)	0.96 (0.47-1.98)	0.9091	0.90 (0.49-1.66)	0.7423
GA + AA	121	145	0.93 (0.68-1.27)	0.6412	116	0.99 (0.71-1.39)	0.9701	0.96 (0.73-1.27)	0.7811
G allele	456 (76.8)	575 (77.9)	1		438 (76.8)	1		1	
A allele	138 (23.2)	163 (22.1)	0.94 (0.72-1.21)	0.6192	132 (23.2)	1.00 (0.76-1.31)	0.9763	0.96 (0.76-1.21)	0.7444

*P -value* ^*a,b,c*^ represents the non-HCC, HCC, and chronic HBV infection patients compared to the healthy control groups adjusted for age, gender, smoking, and drinking by logistic regression analysis. The two-sided *χ*^2^ test or Fisher's exact test was used in allele distribution comparison. CI, confidence interval; ECM, extracellular matrix; HBV, hepatitis B virus; HCC, hepatocellular carcinoma; OR, odds ratio; SNP, single nucleotide polymorphism.

**Table 5:** Genotype and allele frequencies of SNPs in the *ECM1* gene in chronic HBV infection subgroups.

	CHB	LC	CHB *vs.* LC		HCC	LC *vs.* HCC		Non-HCC	Non-HCC vs. HCC	
	*N* (%)	*N* (%)	OR (95% CI)^a^	*P -value* ^*a*^	*N* (%)	OR (95% CI)^b^	*P -value* ^*b*^	*N* (%)	OR (95% CI)^c^	*P*-value^c^
*Rs3834087 genotype and allele*										
Detected number	*N* = 104	*N* = 266(%)			*N* = 286(%)			*N* = 370(%)		
GAG/GAG	96 (92.3)	234 (88.0)	1		243 (85.0)	1		330 (89.2)	1	
GAG/-	8 (7.7)	31 (11.7)	1.63 (0.72-3.68)	0.2442	43 (15.0)	1.24 (0.75-2.05)	0.4121	39 (10.5)	1.40 (0.87-2.25)	0.1622
-/-	0 (0.0)	1 (0.4)	1.00 (0.99-1.01)	1.0000	0 (0.0)	1.00 (0.99-1.00)	0.4922	1 (0.3)	1.00 (0.99-1.00)	1.0000
GAG/- + -/-	8	32	1.67 (0.74-3.78)	0.2162	43	1.19 (0.72-1.97)	0.4884	40	1.36 (0.85-2.18)	0.1963
GAG allele	200 (96.2)	499 (93.8)	1		529 (92.5)	1		699 (94.5)	1	
Del allele	8 (3.8)	33 (6.2)	1.65 (0.75-3.64)	0.2082	43 (7.5)	1.23 (0.77-1.97)	0.3894	41 (5.5)	1.39 (0.89-2.16)	0.1472

*Rs3754217 genotype and allele*										
Detected number	*N* = 104	*N* = 265			*N* = 284			*N* = 369		
GG	61 (58.7)	149 (56.2)	1		146 (54.1)	1		210 (56.9)	1	
GT	37 (35.6)	97 (36.6)	1.15 (0.70-1.88)	0.5791	114 (40.1)	1.18 (0.82-169)	0.3693	134 (36.3)	1.20 (0.86-1.68)	0.2749
TT	6 (5.8)	19 (7.2)	1.35 (0.51-3.56)	0.5512	24 (8.5)	1.13 (0.59-2.19)	0.7068	25 (6.8)	1.24 (0.67-2.29)	0.4901
GT + TT	43	116	1.18 (0.74-1.88)	0.4592	138	1.17 (0.83-1.65)	0.3642	159	1.21 (0.88-1.66)	0.2381
G allele	159 (76.4)	395 (74.5)	1		406 (71.5)	1		554 (75.1)	1	
T allele	49 (23.6)	135 (25.5)	1.11 (0.76-1.61)	0.5891	162 (28.5)	1.17 (0.89-1.53)	0.2559	184 (24.9)	1.20 (0.94-1.54)	0.1449

*Rs3737240 genotype and allele*										
Detected number	*N* = 104	*N* = 265			*N* = 285			*N* = 369		
CC	62 (59.6)	162 (61.1)	1		169 (59.3)	1		224 (60.7)	1	
CT	38 (36.5)	89 (33.6)	0.95 (0.58-1.55)	0.8401	100 (35.1)	0.961 (0.74-1.53)	0.7572	127 (34.4)	1.03 (0.73-1.44)	0.8821
TT	4 (3.8)	14 (5.3)	1.35 (0.42-4.27)	0.6151	16 (5.6)	1.04 (0.49-2.24)	0.9132	18 (4.9)	1.12 (0.55-2.29)	0.7623
CT + TT	42	103	0.90 (0.62-1.59)	0.9679	116	1.06 (0.75-1.50)	0.7546	145	1.04 (0.75-1.43)	0.8229
C allele	162 (77.9)	413 (77.9)	1		438 (76.8)	1		575 (77.9)	1	
T allele	46 (22.1)	117 (22.1)	0.99 (0.68-1.47)	0.9914	132 (23.2)	0.93 (0.70-1.23)	0.6223	163 (22.1)	1.06 (0.73-1.55)	0.7585

*Rs13294 genotype and allele*										
Detected number	*N* = 104	*N* = 265			*N* = 285			*N* = 369		
GG	62 (59.6)	162 (61.1)	1		169 (59.3)	1		224 (60.7)	1	
GA	38 (36.5)	89 (33.6)	0.95 (0.58-1.55)	0.8401	100 (35.1)	1.06 (0.74-1.53)	0.7572	127 (34.4)	1.03 (0.73-1.44)	0.8822
AA	4 (3.8)	14 (5.3)	1.35 (0.42-4.27)	0.6145	16 (5.6)	1.04 (0.49-2.24)	0.9127	18 (4.9)	1.12 (0.55-2.29)	0.7615
GA + AA	42	103	0.99 (0.62-1.59)	0.9682	116	1.06 (0.75-1.50)	0.7550	145	1.04 (0.75-1.43)	0.8230
G allele	162 (77.9)	413 (77.9)	1		438 (76.8)	1		575 (77.9)	1	
A allele	46 (22.1)	117 (22.1)	1.00 (0.68-1.47)	0.9911	132 (23.2)	1.06 (0.80-1.41)	0.6682	163 (22.1)	1.06 (0.82-1.38)	0.6462

*P*-value^a,b,c^ represents the comparison adjusted for age, gender, smoking, and drinking by logistic regression analysis. The two-sided *χ*^2^ test or Fisher's exact test was used in allele distribution comparison. CHB, chronic hepatitis B; CI, confidence interval; ECM, extracellular matrix; HBV, hepatitis B virus; HCC, hepatocellular carcinoma; LC, liver cirrhosis; OR, odds ratio; SNP, single nucleotide polymorphism.

**Table 6:** Haplotype distributions between healthy controls and chronic HBV infection patients.

Haplotype	Frequency		*χ* ^2^	*P*	OR (95% CI)
	Healthy controls (%)	Chronic HBV infection (%)			
*rs3754217-rs3737240*					
G-C	397 (67.7)	931 (71.4)	8.47	*0.0373*	1
G-T	22 (3.8)	28 (2.1)			*0.54 (0.31-0.96)*
T-C	52 (8.9)	80 (6.1)			*0.64 (0.44-0.93)*
T-T	115 (19.6)	265 (20.3)			0.99 (0.77-1.27)

^*∗*^ *rs3834087-rs3754217-rs3737240*					
G-G-C	395 (67.2)	931 (71.4)	17.2	*0.0086*	1
G-G-T	22 (3.7)	28 (2.6)			*0.54 (0.31-0.96)*
G-T-C	3 (0.5)	4 (0.4)			0.55 (0.12-2.49)
G-T-T	115 (19.6)	259 (19.8)			0.95 (0.74-1.22)
D-G-C	4 (0.7)	0 (0.0)			—
D-T-C	49 (8.3)	76 (5.8)			*0.65 (0.45-0.96)*
D-T-T	0 (0.0)	6 (0.3)			—

^*∗*^rs3834087's GAG allele was simply marked with G; DEL allele was simply marked with D. CI, confidence interval; HBV, hepatitis B virus; OR, odda ratio.

**Table 7:** Haplotype distributions between healthy controls and non-HCC patients.

Haplotype	Frequency		*χ* ^2^	*P*	OR (95% CI)
	Healthy controls (%)	Non-HCC (%)			
^*∗*^ *rs3834087-rs3754217*					
G-G	416 (70.7)	554 (75.1)	10.42	*0.0153*	1
G-T	119 (20.2)	144 (19.5)			0.91 (0.69-1.20)
D-G	4 (0.7)	0 (0.0)			1.00 (0.99-1.02)
D-T	49 (8.3)	40 (5.4)			*0.61 (0.40-0.95)*

*rs3754217-rs3737240*					
G-C	398 (67.7)	536 (72.8)	10.42	*0.0153*	1
G-T	22 (3.7)	17 (2.3)			0.57 (0.29-1.11)
T-C	53 (9.0)	37 (5.0)			*0.51 (0.33-0.81*)
T-T	115 (19.6)	146 (19.8)			0.94 (0.72-1.25)

^*∗*^rs3834087's GAG allele was simply marked with G; DEL allele was simply marked with D. CI, confidence interval; HCC, hepatocellular carcinoma; OR, odds ratio.

## Data Availability

The data are available from the corresponding author on reasonable request.

## Abbreviations

SNPs: Single nucleotide polymorphisms
*ECM1*: Extracellular matrix protein 1
CHB: Chronic hepatitis B
LC: Liver cirrhosis
HCC: Hepatocellular carcinoma
HBV: Hepatitis B virus
ECM: Extracellular matrix
HSCs: Hepatic stellate cells
HCV: Hepatitis C virus
HIV: Human immunodeficiency virus
UTR: Untranslated regions
LD: Linkage disequilibrium
TFBS: Transcription factor binding site
PCR: Polymerase chain reaction
H-WE: Hardy–Weinberg equilibrium
ORs: Odds ratios
CIs: Confidence intervals
LSM, kPa: Liver stiffness measurement.

## Supplementary Materials

Supplementary Materials Supplemental Table 1. The liver stiffness measurement (LSM, kPa) score's distribution in FibroScan of rs3834087 and rs3754217 polymorphisms in chronic hepatitis B (CHB) group patients. Supplemental Table 2. The Child-Pugh scores and level's distribution of rs3834087 and rs3754217 polymorphisms in liver cirrhosis.
