# Supplementary material for: Associations between Extracellular Matrix Protein 1 Gene Polymorphism and Progression of Liver Disease
Source: Genet Res (Camb). 2022 Oct 14;2022:9304264. doi: 10.1155/2022/9304264 (PMC9586790; doi:10.1155/2022/9304264)
Supplement: Supplementary Materials — Supplemental Table 1. The liver stiffness measurement (LSM, kPa) score's distribution in FibroScan of rs3834087 and rs3754217 polymorphisms in chronic hepatitis B (CHB) group patients. Supplemental Table 2. The Child-Pugh scores and level's distribution of rs3834087 and rs3754217 polymorphisms in liver cirrhosis. [file 9304264.f1.doc]

**Supplemental Table 1**. The liver stiffness measurement (LSM, Kpa) score’s distribution in FibroScan of rs3834087 and rs3754217 polymorphisms in chronic hepatitis B (CHB) group patients.

| Genotype | LSM | ***P-value*** |
| --- | --- | --- |
| Rs3834087 |  |  |
| GAG/GAG | 10.85 (8.25, 14.1) | 1.0000 |
| GAG/- | 11.65 (8.15, 14.33) | 0.8472 |
| Rs3754217 |  |  |
| GG | 11.35 (9.6, 16.6) | 1.0000 |
| GT | 8.85 (7.60, 12.58) | ***0.0113*** |
| TT | 10.7 (8.15, 12.8) | 0.5234 |
| GT+TT | 10.9 (58.25, 14.1) | ***0.0118*** |

The rank sum test was used to evaluate the data among the groups.

**Supplemental Table 2**. The Child-Pugh scores and level’s distribution of rs3834087 and rs3754217 polymorphisms in liver cirrhosis.

| Genotype |  |  |  |  |
| --- | --- | --- | --- | --- |
| Rs3834087 | GAG/GAG | GAG/- | -/- | ***P-value*** |
| Child-Pugh Score | 9 (7,11) | 9 (7,12) |  | 0.4212 |
| Child-Pugh Level |  |  |  | 0.2348 |
| A | 53 (22.7) | 4 (12.9) | 0 (0.0) |  |
| B | 83 (35.6) | 16 (51.6) | 0 (0.0) |  |
| C | 97 (41.6) | 11 (35.5) | 1 (100) |  |
| Rs3754217 | GG | GT | TT |  |
| Child-Pugh Score | 9 (7,11) | 9 (7,11) | 9 (6,1) |  |
| Child-Pugh Level |  |  |  | 0.8767 |
| A | 31 (20.8) | 20 (20.6) | 3 (33.3) |  |
| B | 52 (34.9) | 39 (40.2) | 7 (38.9) |  |
| C | 66 (44.3) | 38 (39.2) | 5 (27.8) |  |

The rank sum test used to evaluate the continuous variable and the χ2 test was used for grouped variables comparison.
